# First Detection of West Nile Virus (WNV) Lineage 2 in Mosquitoes in the Republic of Kosovo

**DOI:** 10.1155/tbed/3208806

**Published:** 2025-06-24

**Authors:** Ina Hoxha, Betim Xhekaj, Nesade Muja-Bajraktari, Karin Sekulin, Maria S. Unterköfler, Lisa Schlamadinger, Tanto Situmorang, Hans-Peter Fuehrer, Adelheid G. Obwaller, Jeremy V. Camp, Julia Walochnik, Kurtesh Sherifi, Edwin Kniha

**Affiliations:** ^1^Center for Pathophysiology, Infectiology and Immunology, Medical University Vienna, Vienna, Austria; ^2^Faculty of Agriculture and Veterinary, University of Prishtina, Prishtina, Kosovo; ^3^Armaments and Defence Technology Agency, Federal Ministry of Defence, Vienna, Austria; ^4^Department of Biological Sciences and Pathobiology, University of Veterinary Medicine Vienna, Vienna, Austria; ^5^Division of Science, Research and Development, Federal Ministry of Defence, Vienna, Austria; ^6^Center for Virology, Medical University Vienna, Vienna, Austria

**Keywords:** *Culex*, Kosovo, monitoring, phylogeny, West Nile virus

## Abstract

West Nile virus (WNV, family *Flaviviridae*) is the most geographically widespread arbovirus affecting humans. It circulates between wild birds and mosquitoes, while humans and horses are dead-end hosts. In recent years, several outbreaks have been reported from European countries, including the Balkan Peninsula. In the Republic of Kosovo, a southern Balkan country, data on WNV are scarce, and neither mosquito monitoring nor WNV surveillance is established. To address this gap, we aimed to assess a first monitoring approach that should set the basis and support future large-scale activities in the country. Mosquito sampling was performed from May to September 2022 in a peri-urban area in the western part of the capital city Prishtina, Republic of Kosovo. Collected mosquitoes were pooled, homogenized, and total nucleic acid was extracted. A WNV-DENV-ZIKV-specific multiplex RT-qPCR was applied, and WNV-positive samples were confirmed by RT-PCR and whole-genome sequencing. Of 44 screened pools, one pool molecularly identified as *Culex pipiens* f. *pipiens* was positive for WNV RNA. Subsequent sequencing revealed WNV lineage 2, and phylogenetic analysis included our sample in a monophyletic clade consisting mostly of sequences from southeastern Europe. This finding represents the first detection of WNV in mosquitoes in Kosovo, and provides crucial baseline data for future vector-borne disease monitoring, and control efforts in Kosovo.

## 1. Introduction

Mosquitoes (Diptera: Culicidae) are key vectors for many vector-borne pathogens, including West Nile virus (“WNV,” *Orthoflavivirus nilense*, family *Flaviviridae*), which is the most geographically widespread member of the Japanese encephalitis virus (JEV) complex, being endemic on all continents except Antarctica [1]. WNV circulates (i.e., enzootic maintenance) between wild birds and mosquitoes, most importantly species of the genus *Culex*, while infected humans and horses represent dead-end hosts that may show symptoms [2]. In humans, about 80% of infections are asymptomatic, and about 20% result in mild febrile illness (West Nile fever, “WNF”). A neuroinvasive disease (“West Nile neuroinvasive disease,” WNND) may develop after infections with WNV genetic lineages 1 or 2, which are focally endemic in southern, central, and eastern Europe [3–5].

In Europe, WNV circulation typically shows annual amplification with two distinct cycles, either rural with wetland birds and ornithophilic mosquitoes or urban involving synanthropic or domestic birds and ornitho-/anthropophilic mosquitoes, mainly *Cx. pipiens* f. *pipiens* or *Cx. pipiens* f. *molestus* [6–8]. The presence of WNV in the Balkan Peninsula has been increasingly documented, with several outbreaks reported among humans or animals in Croatia [9], Bosnia and Herzegovina [10], Serbia [11], Albania [12], and Greece [13]. Outbreaks in Balkan countries have been confirmed or suspected to be associated with infections of WNV lineage 2, first isolated in the sub-Saharan African country Uganda, where it is also endemic.

In the Republic of Kosovo, a central Balkan country bordered by Montenegro, Serbia, North Macedonia, and Albania, data on WNV are scarce. The first clinical human cases were reported in 2012, and between 2013 and 2015, another 14 infected people were identified [14]. The latest outbreak of WNF in Kosovo was in 2018, with 14 human cases, including three deaths [15]. Retrospective analyses of WNV from positive patients suggested at least two recent and distinct introductions of WNV lineage 2 into Kosovo from neighboring countries [16]. Additionally, the circulation of WNV in horses and birds has been serologically confirmed [17]. *Culex pipiens* s.l. was observed to be the most abundant mosquito species, preferring urban over rural environments in a nationwide entomological survey in Kosovo [18]. However, WNV has not been detected in a small number of mosquitoes collected in 2018–2019 [17].

Until now, neither regular seasonal mosquito monitoring nor WNV surveillance has been established in Kosovo. To address this gap, we aimed to assess a first monitoring approach that should set the basis and support future large-scale activities in the country. Therefore, adult mosquitoes were seasonally sampled in an urban area of the highest populated capital city, Prishtina.

## 2. Material and Methods

### 2.1. Mosquito Sampling and Identification

**Figure 1:**
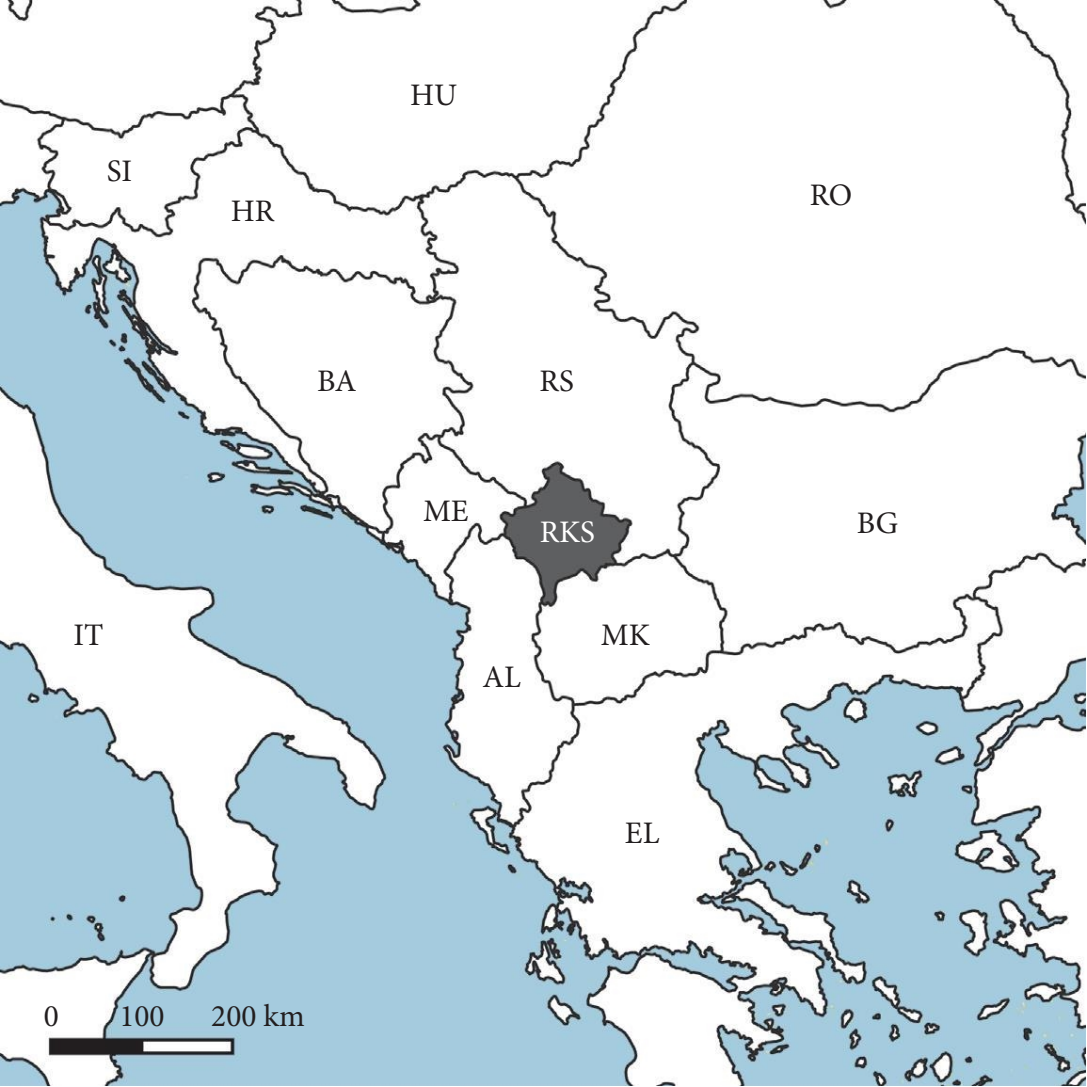
Geographical position of the Republic of Kosovo (dark grey) in the Balkan Peninsula.

A Biogents BG-Sentinel trap baited with CO_2_ was operated for 24 h each week from May to September 2022 in a peri-urban area in the western part of the capital city Prishtina, Republic of Kosovo (Figure 1). Generally, a 7-day trapping interval to make data collection uniform was applied. On some occasions of heavy rain, trapping periods (24 h) were shifted to the next day. Female specimens were morphologically identified based on the identification keys of Becker et al. [19], sorted and pooled by date, species, and sex, and immediately frozen for further analyses.

### 2.2. Nucleic Acid Extraction

Pooled specimens (433 specimens/44 pools) were homogenized in 500 µL Dulbecco's Modified Eagle Medium supplemented with 20% bovine serum albumin, 1% penicillin/streptomycin, 10 µg/mL gentamicin, and 0.25 µg/mL amphotericin B (all from Gibco, Thermo Fischer Scientific, Waltham, MA, USA). Two metal beads (3 mm diameter) were added to each 2.0 mL tube, and the sample was homogenized with a TissueLyser bead mill (QIAGEN GmbH, Hilden, Germany) for 1 min of shaking at 30 Hz. The homogenate was cleared via centrifugation in a 4°C benchtop centrifuge for 5 min at 14,000 rpm. For whole nucleic acid extraction, 200 µL supernatant was taken from mosquito pools. Extraction was performed automatically on the KingFisher Apex Purification System using the innuPREP AniPath DNA/RNA kit (iST Innuscreen, Berlin, Germany).

### 2.3. Detection of WNV RNA

A WNV-DENV-ZIKV specific multiplex RT-qPCR was applied using the primers and probes for WNV: WNV-8-F: 5′-CGCCTGTGTGAGCTGACAAA-3′, WNV-118-R: 5′-GCCCTCCTGGTTTCTTAGACATC-3′, WNV-67-P: 5′-FAM-TGCGAGCTGTTTCTTAGCACGA-BHQ1-3′ [20]; for DENV: DENV-F: 5′-GGAAGTAGAGCAATATGGTACATGTG-3′, DENV-R: 5′-CCGGCTGTGTCATCAGCATAYAT-3′, DENV-P: 5′-HEX-TGTGCAGTCC TTCTCCTTCCACTCCACT-BHQ1-3′ [21]; and for ZIKV: ZIKV-F: 5′-CCGCTGCCCAACACAAG-3′, ZIKV-R: 5′-CCACTAACG TTCTTTTGCAGACAT-3′, ZIKV-P: 5′-Cy5-AGCCTACCTTGACAAGCAATCAGACACTCAA-BHQ2-3′ [22]. Positive control reagents for WNV, DENV serotype 1–4, and ZIKV were used from Viasure (Certest Biotec, Zaragoza, Spain). Multiplex RT-qPCR was performed with Luna Probe One-Step RT-qPCR Kit (New England BioLabs, Ipswich, MA, USA) in an LC480 II Light Cycler PCR System (Roche, Vienna, Austria) according to the following program: one cycle of reverse transcription at 55°C for 10 min, one cycle of initial denaturation at 95°C for 1 min, 45 cycles of denaturation at 95°C for 10 s, and extension at 60°C for 30 s with fluorescence signal acquisition.

WNV-positive samples were confirmed by a universal JEV-group PCR, using a specific oligonucleotide primer pair designed on the nonstructural protein 5 (NS5) and 3′-untranslated regions (UTR) of WNV (forward primer: 5′-GARTGGATGACVACRGAAGACATGCT-3′ and reverse primer: 5′-GGGGTCTCCTCTAACCTCTAGTCCTT-3′) employing the QIAGEN OneStep RT-PCR Kit (Qiagen, Hilden, Germany) [23]. PCR products were subjected to electrophoresis in 2% agarose gels stained with GelRedNucleic Acid Gel Stain (Biotium, Inc., Hayward, CA, USA). For further sequencing, a tiled-amplicon approach was used to amplify the complete virus coding region with partial 3′- and 5′-untranslated regions. The RNA was reverse transcribed into cDNA with LunaScript RT SuperMix (New England BioLabs). Nonoverlapping primer pairs were divided into two pools targeting three amplicons each (Supporting Information 1: Appendix S1), and PCR was performed with Q5 hot start high-fidelity DNA polymerase. The resulting amplicons were pooled, and the sequencing library was prepared with the Nextera XT kit (Illumina Austria GmbH, Vienna, Austria). The library was sequenced on an Illumina MiSeq with V2 chemistry, acquiring 2 × 150 bp paired-end reads. Sequencing adapters were removed with the onboard demultiplexing tool, reads were quality filtered with fastp (v0.23.4), and then mapped to the reference genome (NC_001563) with minimap2 (v2.26) (average sequencing depth 26 k). The program iVar (v1.4.2) was used to remove primers and build the consensus.

### 2.4. *Cx. pipiens* Complex Differentiation

To differentiate *Cx. pipiens* forms from *Cx. torrentium*, partial amplification of a polymorphic gene locus (second intron of the acetylcholinesterase-2, ace-)2 was performed using primers ACEpip, ACEpall, ACEtorr, and B1246s [24]. PCR reactions were performed using 3 μL whole nucleic acid template with 2× EmeraldAmp GT PCR Master Mix (Takara Bio Europe AB, Göteborg, Sweden) in a final volume of 25 µL with an Eppendorf Mastercycler (Eppendorf AG, Hamburg, Germany). Cycling conditions were as follows: 5 min at 94°C, 35 cycles of 30 s at 94°C, 30 s at 55°C, 1 min at 72°C with a final elongation of 5 min at 72°C. PCR products were separated using gel electrophoresis targeting 634 bp (*Cx. pipiens* forms) and 512 bp (*Cx. torrentium*) DNA fragments.

Further, differentiation of *Cx. pipiens* f. *pipiens* and *Cx. pipiens* f. *molestus* by conventional PCR was based on partial CQ11 sequences and performed using primers CQ11F2, pip CQ11R, and mol CQ11R [25]. PCR reactions were performed as mentioned above. Cycling conditions were as follows: 5 min at 94°C, 40 cycles of 30 s at 94°C, 30 s at 54°C, 40 s at 72°C with a final elongation of 5 min at 72°C. PCR products were visualized using gel electrophoresis targeting 185 bp (*Cx. pipiens* f. *pipiens*) and 241 bp (*Cx. pipiens* f. *molestus*) DNA fragments.

### 2.5. Phylogenetic Analysis of WNV

The complete coding region of WNV was compared to reference sequences obtained from GenBank (Supporting Information 2: Table S1). Reference sequences were selected to be representative of WNV lineage 2 in Europe, using two outgroups from Africa (EF429198 and DQ318019). Additionally, all known sequences from Kosovo (four from humans in 2018, MZ190464-MZ190467) were included. The sequences were aligned using MAFFT (v.7.515) FFS-NS-2 fast progressive alignment, and maximum likelihood phylogenies were inferred over 1000 ultrafast bootstraps in iqtree2 (v.2.2.0.3) using the substitution model TIM2+F+R2 determined by the model finder.

## 3. Results

**Figure 2:**
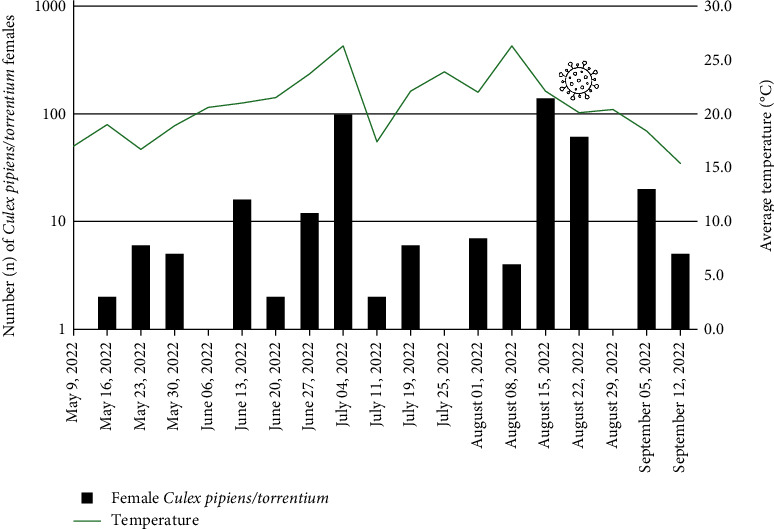
Weekly *Cx. pipiens* s.l. activity at the study site. WNV positive pool indicated by virus symbol. Temperature data were obtained from the Hydrometeorological Institute of Kosovo.

In total, 433 mosquitoes were captured on 19 trap-nights performed weekly from May 9, 2022 to the end of September 12, 2022. First mosquito activity was noted on May 16, and two seasonal peaks of activity were recorded in early July and late August, respectively (Figure 2). Of all trapped specimens, 392 were females and 41 were males. Females of three mosquito species were morphologically identified, namely 386 specimens as *Cx. pipiens*/*torrentium*, five specimens as *Culiseta longiareolata*, and one as *Anopheles maculipennis* s.l.

Of 44 screened pools, one pool comprising 20 females *Cx. pipiens* s.l. collected on August 22, 2022, was positive for WNV RNA. Molecular species identification revealed that the WNV RNA-positive mosquito pool only comprised *Cx. pipiens* f. *pipiens* specimens (Supporting Information 3: Figure S1 and Supporting Information 4: Figure S2). Sequencing revealed the closest identity to WNV lineage 2, showing 99.57–99.28% identity with reference sequences from Hungary (e.g., PP212880 and PP212882), and 99.13% with sequences from Greece (MN481594), Serbia (KT757323), Bulgaria (MT341472), and Belgium (MH021189).

**Figure 3:**
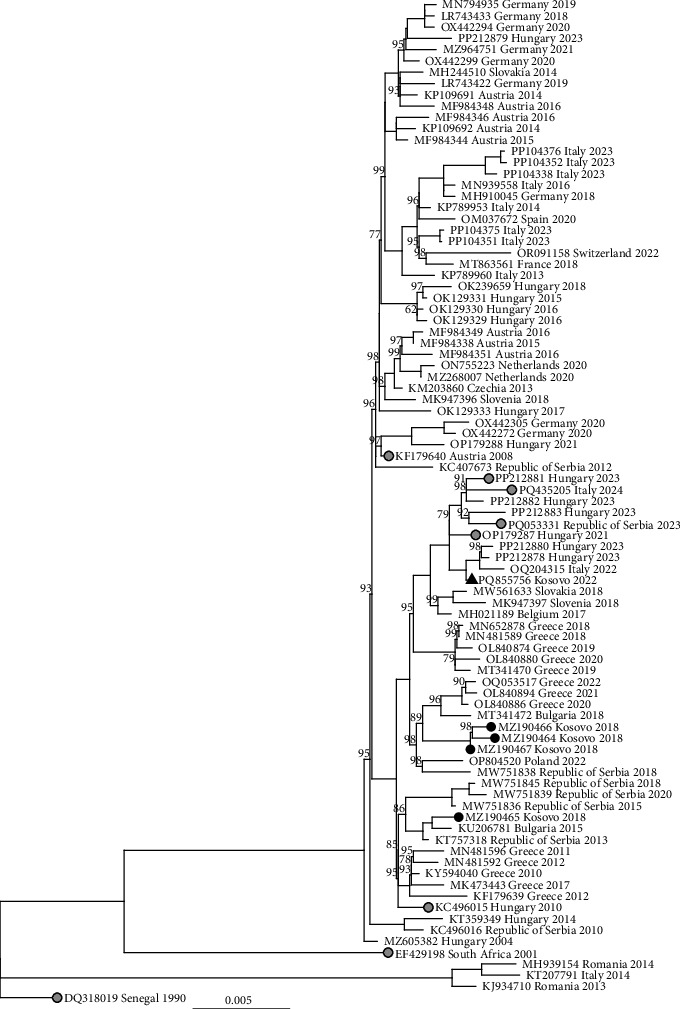
Maximum likelihood phylogeny of selected West Nile virus lineage 2 strains, including the sequence from a pool of *Cx. pipiens* f. *pipiens* captured in Prishtina, Kosovo, 2022. The consensus tree was inferred over 1000 ultrafast bootstrap replicates in IQTree2 using the TIM2+F+R2 substitution model on an alignment of the complete coding sequence. Bootstrap supports (>60%) are given above branches. Sequences originating from humans in Kosovo, 2018, are highlighted with black circles (previous studies) and from a pool of mosquitoes in Kosovo, 2022, with a black triangle (2022). Sequences indicated by grey circles were used as references to compare sequence similarity (Supporting Information 1: Appendix S1). The scale bar shows substitutions per site over the 10,302 bp open reading frame.

The maximum likelihood tree demonstrated that the sequence formed a monophyletic clade with sequences from Hungary in 2021 to 2023 (OP179287 and PP202880, respectively), Italy in 2022 to 2024 (OQ204315 and PQ453205, respectively), and the Republic of Serbia in 2023 (PQ053331) (Figure 3). The sequence had the highest nucleotide identity to these strains (99.56%−99.75%), differing at 26–45 sites, resulting in 3–9 amino acid substitutions (Supporting Information 4: Table S1); two of which (E:K689R and NS1:I1035V) were specific to this clade on the phylogenetic tree and not found in other sequences in GenBank (Supporting Information 5: Table S2, Supporting Information 6: Table S3, Supporting Information 7: Table S4, and Supporting Information 8: Table S5). One nonsynonymous mutation (NS3:I1787V) was found only in two sequences described from human patients from Hungary, 2023 (PP212878 and PP212880), and one sequence from *Cx. pipiens* mosquitoes captured in Sicily, Italy, 2022 (OQ204315) (Supporting Information 5: Table S2, Supporting Information 6: Table S3, and Supporting Information 9: Table S6). One mutation (C:A121V) was also found within this group; however, it appears to be homoplastic and was also in sequences identified in Italy in 2023 (PP104351 and PP104375, Figure 3). All sequences from Kosovo are within a monophyletic clade of WNV lineage 2 consisting mostly of sequences from southeastern Europe compared to another monophyletic clade with sequences from central and western Europe (Figure 3). However, the sequence from *Culex* mosquitoes in 2022 differed by 63–76 nucleotides (99.26%−99.39% nucleotide identity) and 12–16 amino acid mutations in the open reading frame compared to previously identified WNV in humans in Kosovo, 2018 (Supporting Information 4: Table S1, Supporting Information 5: Table S2, and Supporting Information 6: Table S3). For example, the 2022 sequence did not have the NS3:K1720R mutations found throughout the previously described “southeast European subclade I,” nor did it have the other nonsynonymous mutations previously described from Kosovo (e.g., NS3:D1906E, NS3:V2087I, NS5:R2544K, NS5:K2988N) (Supporting Information 7: Table S4, Supporting Information 8: Table S5, Supporting Information 9: Table S6, and Supporting Information 10: Table S7).

## 4. Discussion

While several human WNV infections have been described before in Kosovo [14], the first confirmed human infections were reported to ECDC in 2018 [26]. Serological evidence and time-resolved phylodynamic analysis suggest that WNV was introduced into Kosovo between 2008 and 2012 [16, 17]. After an initial serological survey of horses in 2010, there was no further evidence of WNV infections in Kosovo for several years [12]. The first serological evidence in equines and birds in Kosovo was reported in 2019, with samples collected from January 2018 to June 2019 [17]. A retrospective analysis of archived human sera was able to retrieve whole WNV genomes from infected patients in 2018 [16]. Although mosquitoes in Kosovo have been investigated for WNV without positivity [17], the current study is the first identification of WNV lineage 2 in mosquitoes from Kosovo. This confirms that WNV is endemic in Kosovo.

Since the first detection of WNV lineage 2 in Hungary in 2004 [3] and its rapid spread across Europe in 2008–2009 [4, 26], WNV lineage 2 has been the predominant lineage in Europe. In the following years, various countries have reported seasonal outbreaks of WNV of varying severity [27, 28]. Notably, in 2018 many European countries, including Balkan countries such as Greece [29] or Serbia [30], experienced the largest outbreak of WNV in recorded history. The increasing incidence of WNF and WNND has sparked mandatory monitoring and reporting requirements in many countries and at the EU-level, which rely on integrated surveillance of humans, animals, and vectors [6]. Here, we report the first approach of a small-scale seasonal monitoring, and the first detection of WNV lineage 2 from mosquitoes in the country. Despite presenting data from a single trapping season of a single location, our results highlight the urgency to establish longer-term and larger-scale mosquito monitoring in Kosovo.

While the virus may infect mosquito species of various genera, members of the genus *Culex* are the most important vectors. In Europe, the *Cx. pipiens* complex, including the ornithophilic *Cx. pipiens* f. *pipiens*, the mammalophilic *Cx. pipiens* f. *molestus*, their hybrid forms, as well as the sibling species *Cx. torrentium* are confirmed vectors [31–33]. We identified a pool of *Cx. pipiens* f. *pipiens* to be infected with WNV. This ecotype is regularly reported to be associated with rural environments and preferentially feeding on birds [34–36]. However, Martínez-de la Puente et al. [35] identified a substantial number of mammalian bloodmeals in *Cx. pipiens* f. *pipiens* from Spain. Our sampling location was an urban environment comprising small private households with gardens and apartment complexes. A Kosovo-wide mosquito survey by Muja-Bajraktari et al. [18] also identified *Cx. pipiens* s.l. as the most recorded species, being highly abundant in urban areas.

Urban areas provide diverse microhabitats that support the establishment and maintenance of mosquitoes [37]. In our sampling location, private households may keep chicken, goats, sheep, or other small livestock, providing blood sources for adult female mosquitoes in proximity to humans. Additionally, a large military camp is located close to our sampling location, and thus, personnel stationed there might be at risk of being infected with WNV. For example, Austrian soldiers employed in the Kosovo showed elevated antibody levels against sand fly-borne phleboviruses [38]. Moreover, recently, Emmerich et al. [16] identified a human WNV seroprevalence of 1.55% among 453 randomly selected sera from a hospital in Kosovo and were able to sequence whole viral genomes from four patients.

The current phylogeographic appearance of WNV is complex. While WNV has been maintained in a given geographic region over the years, occasional transfers and introductions between regions have been documented [16, 28]. Emmerich et al. [16] identified at least two potential introduction events of WNV from Serbia and Bulgaria to Kosovo, possibly around 2010–2012, which aligns with evidence from serological surveys [17]. Our sample from 2022 was in a clade with sequences from the “southeast European clade” [39], including the Kosovo 2018 samples [16], but the sequences were more similar to sequences identified in Hungary and Italy from 2021 to 2023, sharing specific nonsynonymous mutations. The biological significance of these mutations is unknown, but it has been established that WNV lineage 2 evolution in Europe is driven by strong purifying selection, and therefore, the mutations are likely neutral, and their fixation is likely the result of random drift [40]. Therefore, although we did not attempt to isolate the virus, it is unlikely to have a phenotype different from other WNV-lineage 2 circulating in Europe, as none of the mutations have known virulence/attenuation properties [41]. Very few studies have been performed comparing WNV pathogenicity among contemporary WNV-lineage 2 strains [42]. Most studies have identified virulence-associated mutations by comparing phylogenetically distant strains [43–45]; therefore, we conclude that the strain we characterized here will likely have a similar phenotype to the few previous descriptions of contemporary WNV-lineage 2 in Europe [42, 46]. Our phylogenetic analysis supports the consensus that at least two clades of lineage 2 are currently circulating in Europe based on whole viral genome sequencing [47]. Providing the first identification of WNV in mosquitoes in Kosovo, as well as viral sequence data, enables more precise analyses of WNV phylogeography and will assist future surveillance efforts in the region.

## 5. Conclusion

We report the first detection of WNV lineage 2 in mosquitoes in Kosovo and provide crucial baseline data for future vector-borne disease monitoring and control efforts in Kosovo. With increasing WNF and WNND cases throughout Europe, there is a need for continued surveillance to further establish public health measures and potentially prevent future outbreaks.

## Data Availability

All the data are included in the article.

## Supporting Information

Supporting Information 1 Appendix S1: Whole genome sequencing methods.

Supporting Information 2 Figure S1: Agarose gel of amplified fragments using *Cx. pipiens*-specific primers (P), *Cx. torrentium*-specific primers (T), and a multiplex reaction combining all three primers (P+T). M = DNA stepladder, N = negative control, lane 1 = *Cx. torrentium* DNA not amplified with *Cx. pipiens* specific primers, lane 2 = *Cx. pipiens* DNA amplified with *Cx. pipiens* specific primers, lane 3 = WNV-positive mosquito pool amplified with *Cx. pipiens* specific primers, lane 5 = *Cx. torrentium* DNA amplified with *Cx. torrentium*-specific primers, lane 6 = *Cx. pipiens* DNA not amplified with *Cx. torrentium*-specific primers, lane 7 = WNV-positive mosquito pool not amplified with *Cx. torrentium*-specific primers, lane 9 = *Cx. torrentium* DNA amplified with multiplex reaction combining all three primers, lane 10 = *Cx. pipiens* DNA amplified with multiplex reaction combining all three primers, lane 11 = WNV-positive mosquito pool amplified with multiplex reaction combining all three primers.

Supporting Information 3 Figure S2: Agarose gel of PCR discriminating between *Cx. pipiens* f. *pipiens* and *Cx. pipiens* f. *molestus*. M = DNA stepladder, lane 1 = WNV-positive mosquito pool showing a *Cx. pipiens* f. *pipiens* specific band (185 bp), lane 3 and 5 = *Cx. pipiens* f. *molestus* DNA showing a specific band (241 bp), lanes 2 and 4 = empty.

Supporting Information 4 Table S1: Sequence identity matrix comparing percent nucleotide (lower) and percent amino acid (upper) identity between the selected strains across the complete 10302 nt polyprotein open reading frame.

Supporting Information 5 Table S2: Percent nucleotide identity for each of the gene products made by posttranslational cleavage of the WNV lineage 2 polyprotein compared to a sequence identified in mosquitoes in Kosovo, 2022.

Supporting Information 6 Table S3: Percent amino acid identity for each of the gene products made by post-translational cleavage of the WNV lineage 2 polyprotein compared to a sequence identified in mosquitoes in Kosovo, 2022.

Supporting Information 7 Table S4: Specific nonsynonymous mutations in the structural proteins (anchored capsid, “ancC”; membrane glycoprotein, “M”; envelope glycoprotein, “E”) of the selected West Nile virus lineage 2.

Supporting Information 8 Table S5: Specific nonsynonymous mutations in the nonstructural proteins (NS1, NS2a, NS2b) of the selected West Nile virus lineage 2.

Supporting Information 9 Table S6: Specific nonsynonymous mutations in the non-structural proteins (NS3, NS4a, NS4b) of the selected West Nile virus lineage 2.

Supporting Information 10 Table S7: Specific nonsynonymous mutations in the RNA-dependent RNA polymerase (NS5) of selected West Nile virus lineage 2.
