# Supplementary material for: First Detection of West Nile Virus (WNV) Lineage 2 in Mosquitoes in the Republic of Kosovo
Source: Transbound Emerg Dis. 2025 Jun 24;2025:3208806. doi: 10.1155/tbed/3208806 (PMC12213049; doi:10.1155/tbed/3208806)
Supplement: Supporting Information 1 — Appendix S1: Whole genome sequencing methods. [file 3208806.f1.docx]

**Supporting Information 1: Appendix S1.** Whole genome sequencing methods.

The primers were taken from Pappa, *et al.* (2021) “PCR-based next-generation West Nile virus sequencing protocols” *Molecular and Cellular Probes* 60:101774, with minor modifications indicated by underlined nucleotides in the table below (one nucleotide from the 5’ end of primer WN2-1stupP4b was not included). The cDNA synthesis step used random primers and poly-dT oligonucleotides contained in the LunaScript SuperMix, and not the cDNA primer recommended by Pappa, *et al.* (2021).

| **Product** | **Primer** | **Sequence (5’ 🡪 3’)** | **Pool** |
| --- | --- | --- | --- |
| P1 | WN2-1stupP1 | AGTAGTTCGCCTGTGTGAGCTGA | 1 |
|  | WN2-1stdoP1 | TGCAGTTCCAACACCACCGTTCC | 1 |
| P2 | WN2-1stupP2 | TCTGTTGTGGCTCTAGGGTCGCAG | 2 |
|  | WN2-1stdoP2 | CTCTCTTGGTTGGTCCACYTTGCCTTCA | 2 |
| P3 | WN2-1stupP3 | CCCAGGAGGTCCTTCGCAAGAGGTG | 1 |
|  | WN2-1stdoP3 | ACCAAACGGTCTTTCCAACATACTCAGT | 1 |
| P4 | WN2-1stupP4b | GAGCACAGTGGAAATGAGATCGTTGATG | 2 |
|  | WN2-1stdoP4 | TGACTGGAGTTGTTCGCTCAAGTTCAGGT | 2 |
| P5 | WN2-1stupP5 | TGTGGACGTTGGTGTGTCAGCTCT | 1 |
|  | WN2-1stdoP5 | GTGTGTCCCAGCCTGCRGTRTCATC | 1 |
| P6 | WN2-1stupP6 | TGCATCTACAACATGATGGGAAAGAGAGAG | 2 |
|  | WN2-1stdoP6 | CGCACTGTGCCGTGTGGCTGGT | 2 |

The amplicons were between 1949-2129 base pairs, and were pooled before preparing for sequencing. The library was fragmented with tagmatase according to the NexteraXT protocol, and multiplexed by PCR unique dual index barcoding. The sample was included with other samples as part of routine sequencing in the diagnostic laboratory at the National Reference Center for Arboviruses at the Center for Virology of the Medical University of Vienna.

A large list of previously described WNV lineage 2 sequences from Europe were included in the maximum likelihood phylogenetic analysis, removing duplicated sequences, and manually reducing the number of sequences from the same country from the same year. To compare sequence similarity and describe specific nonsynonymous mutations, twelve additional sequences were selected: a historic WNV-2 (DQ318019, Senegal 1990), and a sequence from Africa that shares the closest common ancestor with the clade(s) of contemporary WNV-2 in Europe (EF429198). We included two of the first WNV-2 that have been continuously circulating in Europe since the first detection in Hungary 2004 (KF179640 Austria 2008 and KC496015 Hungary 2010) and share common ancestors with each of the major clades of the current outbreak. We included the four previously described WNV-2 from Kosovo in humans, 2018 (MZ190464-MW190467), as well as four sequences that share a common ancestor with the sequence determined from 2022 and have the highest sequence identities to this strain.

| **Accession** | **Country** | **Strain** | **Year** | **Source** |
| --- | --- | --- | --- | --- |
| DQ318019 | Senegal | ArD76104 | 1990 | Mosquito |
| EF429198 | South Africa | SA93/01 | 2001 | Human |
| KC496015 | Hungary | 578/10 | 2010 | Horse |
| KF179640 | Austria | 2008_gh | 2008 | Goshawk |
| MZ190464 | Kosovo | Kosovo_1_2018 | 2018 | Human |
| MZ190465 | Kosovo | Kosovo_2_2018 | 2018 | Human |
| MZ190466 | Kosovo | Kosovo_3_2018 | 2018 | Human |
| MZ190467 | Kosovo | Kosovo_4_2018 | 2018 | Human |
| OP179287 | Hungary | HUN_WNV2324_2021 | 2021 | Human |
| PP212881 | Hungary | 4106_2023_WNV_HU | 2023 | Human |
| PQ053331 | Serbia | WNF Belgrade 8912/2023 | 2023 | Mosquito |
| PQ435205 | Italy | S1950_IZSM | 2024 | Human |

*Results*

Table S1. Sequence identity matrix comparing percent nucleotide (lower) and percent amino acid (upper) identity between the selected strains across the complete 10302 nt polyprotein open reading frame.

| NT\AA | DQ318019 | EF429198 | KC496015 | KF179640 | MZ190464 | MZ190465 | MZ190466 | MZ190467 | OP179287 | PP212881 | PQ053331 | PQ435205 | Kosovo_2024 |
| --- | --- | --- | --- | --- | --- | --- | --- | --- | --- | --- | --- | --- | --- |
| DQ318019 |  | 99.4 | 99.4 | 99.6 | 99.2 | 99.3 | 99.3 | 99.3 | 99.4 | 99.5 | 99.3 | 99.3 | 99.4 |
| EF429198 | 97.9 |  | 99.2 | 99.4 | 99.0 | 99.1 | 99.0 | 99.1 | 99.1 | 99.2 | 99.0 | 99.0 | 99.2 |
| KC496015 | 97.7 | 97.3 |  | 99.7 | 99.5 | 99.6 | 99.6 | 99.6 | 99.7 | 99.7 | 99.6 | 99.6 | 99.7 |
| KF179640 | 97.8 | 97.5 | 99.6 |  | 99.5 | 99.6 | 99.6 | 99.6 | 99.7 | 99.8 | 99.6 | 99.6 | 99.7 |
| MZ190464 | 97.4 | 97.0 | 99.3 | 99.3 |  | 99.4 | 99.9 | 99.9 | 99.5 | 99.6 | 99.4 | 99.4 | 99.5 |
| MZ190465 | 97.5 | 97.2 | 99.5 | 99.5 | 99.2 |  | 99.4 | 99.5 | 99.5 | 99.6 | 99.4 | 99.4 | 99.6 |
| MZ190466 | 97.4 | 97.0 | 99.3 | 99.3 | 99.8 | 99.3 |  | 100.0 | 99.6 | 99.7 | 99.5 | 99.5 | 99.6 |
| MZ190467 | 97.5 | 97.1 | 99.5 | 99.4 | 99.9 | 99.4 | 99.9 |  | 99.6 | 99.7 | 99.5 | 99.5 | 99.7 |
| OP179287 | 97.6 | 97.1 | 99.4 | 99.4 | 99.2 | 99.3 | 99.2 | 99.3 |  | 99.9 | 99.7 | 99.7 | 99.8 |
| PP212881 | 97.5 | 97.1 | 99.4 | 99.3 | 99.2 | 99.2 | 99.2 | 99.3 | 99.7 |  | 99.8 | 99.8 | 99.9 |
| PQ053331 | 97.5 | 97.0 | 99.3 | 99.3 | 99.1 | 99.2 | 99.1 | 99.3 | 99.6 | 99.7 |  | 99.6 | 99.7 |
| PQ435205 | 97.4 | 96.9 | 99.2 | 99.2 | 99.0 | 99.1 | 99.1 | 99.2 | 99.5 | 99.7 | 99.5 |  | 99.7 |
| Kosovo_2024 | 97.6 | 97.2 | 99.5 | 99.4 | 99.3 | 99.3 | 99.3 | 99.4 | 99.7 | 99.7 | 99.6 | 99.6 |  |

Table S2. Percent nucleotide identity for each of the gene products made by post-translational cleavage of the WNV lineage 2 polyprotein compared to a sequence identified in mosquitoes in Kosovo, 2022.

|  | C | ancC | prM | pr | M | E | NS1 | NS2a | NS2b | NS3 | NS4a | 2k | NS4b | NS5 |
| --- | --- | --- | --- | --- | --- | --- | --- | --- | --- | --- | --- | --- | --- | --- |
| DQ318019 | 99.05% | 98.64% | 98.20% | 98.19% | 98.22% | 97.67% | 97.54% | 98.41% | 95.67% | 97.74% | 98.93% | 95.65% | 96.35% | 97.57% |
| EF429198 | 98.73% | 98.10% | 98.40% | 98.19% | 98.67% | 97.21% | 97.44% | 97.26% | 96.95% | 97.15% | 98.13% | 94.20% | 96.22% | 97.02% |
| KC496015 | 99.68% | 99.46% | 99.60% | 100% | 99.11% | 99.53% | 99.62% | 99.71% | 98.47% | 99.30% | 99.73% | 98.55% | 98.96% | 99.67% |
| KF179640 | 99.68% | 99.46% | 99.60% | 99.64% | 99.56% | 99.53% | 99.53% | 99.57% | 98.47% | 99.25% | 100% | 98.55% | 99.09% | 99.52% |
| MZ190464 | 99.05% | 98.64% | 99.00% | 99.28% | 98.67% | 99.33% | 99.72% | 99.57% | 98.47% | 99.03% | 99.47% | 100% | 99.35% | 99.30% |
| MZ190465 | 99.68% | 99.46% | 99.40% | 99.64% | 99.11% | 99.27% | 99.24% | 99.42% | 98.22% | 99.19% | 100% | 98.55% | 99.48% | 99.56% |
| MZ190466 | 99.05% | 98.64% | 99.00% | 99.64% | 98.22% | 99.33% | 99.91% | 99.57% | 98.47% | 99.08% | 99.73% | 100% | 99.22% | 99.26% |
| MZ190467 | 99.37% | 98.92% | 99.20% | 99.64% | 98.67% | 99.47% | 99.91% | 99.71% | 98.47% | 99.14% | 99.73% | 100% | 99.35% | 99.41% |
| PQ435205 | 100% | 99.73% | 100% | 100% | 100% | 99.73% | 99.72% | 98.99% | 98.98% | 99.35% | 99.20% | 100% | 99.87% | 99.63% |
| PP212881 | 99.68% | 99.46% | 100% | 100% | 100% | 99.73% | 99.81% | 99.86% | 99.49% | 99.52% | 100% | 100% | 99.74% | 99.59% |
| PQ053331 | 100% | 99.73% | 100% | 100% | 100% | 99.93% | 99.81% | 99.86% | 98.73% | 99.41% | 99.47% | 98.55% | 99.74% | 99.56% |
| OP179287 | 100% | 99.73% | 100% | 100% | 100% | 99.80% | 99.81% | 99.86% | 99.24% | 99.52% | 99.47% | 100% | 99.87% | 99.85% |

Table S3. Percent amino acid identity for each of the gene products made by post-translational cleavage of the WNV lineage 2 polyprotein compared to a sequence identified in mosquitoes in Kosovo, 2022.

|  | C | ancC | prM | pr | M | E | NS1 | NS2a | NS2b | NS3 | NS4a | 2k | NS4b | NS5 |
| --- | --- | --- | --- | --- | --- | --- | --- | --- | --- | --- | --- | --- | --- | --- |
| DQ318019 | 100% | 99.19% | 100% | 100% | 100% | 99% | 99.43% | 100% | 98.47% | 99.52% | 100% | 100% | 98.44% | 99.78% |
| EF429198 | 100% | 99.19% | 100% | 100% | 100% | 99.6% | 99.15% | 99.57% | 98.47% | 99.52% | 100% | 100% | 98.44% | 98.67% |
| KC496015 | 100% | 99.19% | 100% | 100% | 100% | 99.4% | 99.43% | 100% | 99.24% | 99.84% | 100% | 100% | 99.22% | 100% |
| KF179640 | 100% | 99.19% | 100% | 100% | 100% | 99.6% | 99.72% | 100% | 99.24% | 99.68% | 100% | 100% | 99.22% | 100% |
| MZ190464 | 100% | 98.37% | 99.4% | 100% | 98.67% | 99.4% | 99.72% | 100% | 100% | 99.35% | 99.2% | 100% | 100% | 99.56% |
| MZ190465 | 100% | 99.19% | 99.4% | 98.91% | 100% | 99% | 99.43% | 100% | 98.47% | 99.84% | 100% | 100% | 99.61% | 99.89% |
| MZ190466 | 100% | 98.37% | 99.4% | 100% | 98.67% | 99.6% | 99.72% | 100% | 100% | 99.35% | 100% | 100% | 100% | 99.67% |
| MZ190467 | 100% | 98.37% | 99.4% | 100% | 98.67% | 99.8% | 99.72% | 100% | 100% | 99.35% | 100% | 100% | 100% | 99.67% |
| PQ435205 | 100% | 99.19% | 100% | 100% | 100% | 100% | 99.72% | 98.7% | 99.24% | 99.68% | 100% | 100% | 100% | 100% |
| PP212881 | 100% | 99.19% | 100% | 100% | 100% | 100% | 100% | 100% | 100% | 99.84% | 100% | 100% | 100% | 99.89% |
| PQ053331 | 100% | 99.19% | 100% | 100% | 100% | 100% | 100% | 100% | 100% | 99.35% | 100% | 100% | 100% | 99.56% |
| OP179287 | 100% | 99.19% | 100% | 100% | 100% | 100% | 99.43% | 100% | 100% | 99.84% | 99.2% | 100% | 99.61% | 100% |

Table S4. Specific nonsynonymous mutations in the structural proteins (anchored capsid, “ancC”; membrane glycoprotein, “M”; envelope glycoprotein, “E”) of the selected West Nile virus lineage 2

|  | ancC | | | M | E | | | | | | | | | | |
| --- | --- | --- | --- | --- | --- | --- | --- | --- | --- | --- | --- | --- | --- | --- | --- |
| Accession | 114 | 121 | 124 | 274 | 381 | 444 | 449 | 530 | 603 | 629 | 668 | 689 | 726 | 729 | 777 |
| DQ318019 | L | A | V | Q | V | - | I | V | R | P | P | K | T | G | V |
| EF429198 | L | A | V | Q | V | NYST | I | M | G | P | P | K | T | G | V |
| KC496015 | L | A | V | Q | V | NYST | I | M | G | P | P | K | T | G | V |
| KF179640 | L | A | V | Q | V | NYST | I | M | G | P | P | K | T | G | V |
| MZ190464 | M | A | V | P | V | NYST | K | M | G | H | P | K | T | G | V |
| MZ190465 | L | A | L | Q | L | NYST | I | M | G | P | S | K | A | G | V |
| MZ190466 | M | A | V | P | V | NYST | T | M | G | P | P | K | T | A | V |
| MZ190467 | M | A | V | P | V | NYST | T | M | G | P | P | K | T | G | V |
| OP179287 | L | A | V | Q | V | NYST | T | M | G | P | P | R | T | G | V |
| PP212881 | L | A | V | Q | V | NYST | T | M | G | P | P | R | T | G | V |
| PQ053331 | L | A | V | Q | V | NYST | T | M | G | P | P | R | T | G | V |
| PQ435205 | L | A | V | Q | V | NYST | T | M | G | P | P | R | T | G | A |
| This study | L | V | V | Q | V | NYST | T | M | G | P | P | R | T | G | V |

Table S5. Specific nonsynonymous mutations in the non-structural proteins (NS1, NS2a, NS2b) of the selected West Nile virus lineage 2

|  | NS1 | | | | | | | | NS2a | | | NS2b | | | | | |
| --- | --- | --- | --- | --- | --- | --- | --- | --- | --- | --- | --- | --- | --- | --- | --- | --- | --- |
| Accession | 820 | 835 | 837 | 882 | 900 | 990 | 1035 | 1066 | 1196 | 1241 | 1255 | 1405 | 1416 | 1462 | 1490 | 1493 | 1502 |
| DQ318019 | M | K | I | K | K | S | I | I | V | R | A | I | A | M | L | V | Y |
| EF429198 | I | K | I | K | K | S | I | I | V | K | A | I | A | M | L | V | Y |
| KC496015 | M | R | I | K | K | A | I | I | V | R | A | V | A | I | L | I | Y |
| KF179640 | M | R | I | K | K | S | I | I | V | R | A | I | A | I | L | V | Y |
| MZ190464 | M | R | I | K | K | S | I | I | V | R | A | I | A | I | L | I | Y |
| MZ190465 | M | R | I | N | K | S | I | I | V | R | A | I | S | I | P | I | Y |
| MZ190466 | M | R | I | K | K | S | I | I | V | R | A | I | A | I | L | I | Y |
| MZ190467 | M | R | I | K | K | S | I | I | V | R | A | I | A | I | L | I | Y |
| OP179287 | M | R | I | K | R | S | V | L | V | R | A | I | A | I | L | I | Y |
| PP212881 | M | R | I | K | K | S | V | I | V | R | A | I | A | I | L | I | Y |
| PQ053331 | M | R | I | K | K | S | V | I | V | R | A | I | A | I | L | I | Y |
| PQ435205 | M | R | S | K | K | S | V | I | D | R | V | I | A | I | L | I | C |
| This study | M | R | I | K | K | S | V | I | V | R | A | I | A | I | L | I | Y |

Table S6. Specific nonsynonymous mutations in the non-structural proteins (NS3, NS4a, NS4b) of the selected West Nile virus lineage 2

|  | NS3 | | | | | | | | | | NS4a | | NS4b | | | | | | | |
| --- | --- | --- | --- | --- | --- | --- | --- | --- | --- | --- | --- | --- | --- | --- | --- | --- | --- | --- | --- | --- |
| Accession | 1516 | 1519 | 1547 | 1574 | 1720 | 1754 | 1787 | 1906 | 2026 | 2087 | 2149 | 2198 | 2284 | 2287 | 2293 | 2296 | 2305 | 2322 | 2386 | 2515 |
| DQ318019 | K | K | V | W | K | H | I | D | D | V | D | R | N | G | H | A | S | T | V | M |
| EF429198 | K | K | V | W | K | H | I | D | D | V | D | R | N | S | K | A | S | T | V | T |
| KC496015 | R | K | V | W | K | P | I | D | D | V | D | R | S | G | R | T | N | A | V | T |
| KF179640 | R | K | V | W | K | H | I | D | D | V | D | R | N | S | K | T | N | T | V | T |
| MZ190464 | R | K | V | W | R | P | I | E | D | I | E | R | N | G | K | T | N | A | V | T |
| MZ190465 | R | K | V | W | K | P | I | D | D | V | D | R | N | G | K | T | N | A | M | T |
| MZ190466 | R | K | V | W | R | P | I | E | D | I | D | R | N | G | K | T | N | A | V | T |
| MZ190467 | R | K | V | W | R | P | I | E | D | I | D | R | N | G | K | T | N | A | V | T |
| OP179287 | R | K | V | W | K | P | I | D | D | V | D | S | D | G | K | T | N | A | V | T |
| PP212881 | R | K | V | W | K | P | I | D | D | V | D | R | N | G | K | T | N | A | V | T |
| PQ053331 | R | Q | G | L | K | P | I | D | D | V | D | R | N | G | K | T | N | A | V | T |
| PQ435205 | R | K | V | W | K | P | I | D | A | V | D | R | N | G | K | T | N | A | V | T |
| This study | R | K | V | W | K | P | V | D | D | V | D | R | N | G | K | T | N | A | V | T |

Table S7. Specific nonsynonymous mutations in the RNA-dependent RNA polymerase (NS5) of selected West Nile virus lineage 2.

|  | NS5 | | | | | | | | | | | | | | | | | |
| --- | --- | --- | --- | --- | --- | --- | --- | --- | --- | --- | --- | --- | --- | --- | --- | --- | --- | --- |
| Accession | 2544 | 2690 | 2719 | 2747 | 2988 | 3055 | 3061 | 3072 | 3096 | 3111 | 3118 | 3143 | 3145 | 3154 | 3173 | 3192 | 3257 | 3425 |
| DQ318019 | R | T | K | EMY | K | T | VY | R | A | V | G | T | T | MM | K | M | T | DT |
| EF429198 | R | T | K | EMY | K | I | IF | P | A | G | R | P | S | RR | N | M | T | DA |
| KC496015 | R | T | R | EMY | K | T | IY | R | A | V | G | T | T | MM | K | M | T | DT |
| KF179640 | R | T | R | EMY | K | T | IY | R | A | V | G | T | T | MM | K | M | T | DT |
| MZ190464 | K | T | R | EMY | N | T | IY | R | A | V | G | T | T | MM | K | L | T | ET |
| MZ190465 | R | T | R | EMY | K | T | IY | R | P | V | G | T | T | MM | K | M | T | DT |
| MZ190466 | K | T | R | EMY | N | T | IY | R | A | V | G | T | T | MM | K | L | T | DT |
| MZ190467 | K | T | R | EMY | N | T | IY | R | A | V | G | T | T | MM | K | L | T | DT |
| OP179287 | R | T | R | EMY | K | T | IY | R | A | V | G | T | T | MM | K | M | T | DT |
| PP212881 | R | S | R | EMY | K | T | IY | R | A | V | G | T | T | MM | K | M | T | DT |
| PQ053331 | R | T | R | GIC | K | T | IY | R | A | V | G | T | T | MM | K | M | M | DT |
| PQ435205 | R | T | R | EMY | K | T | IY | R | A | V | G | T | T | MM | K | M | T | DT |
| This study | R | T | R | EMY | K | T | IY | R | A | V | G | T | T | MM | K | M | T | DT |
