# Supplementary figures and images for: First Detection of West Nile Virus (WNV) Lineage 2 in Mosquitoes in the Republic of Kosovo
Source: Transbound Emerg Dis. 2025 Jun 24;2025:3208806. doi: 10.1155/tbed/3208806 (PMC12213049; doi:10.1155/tbed/3208806)

## Slide 1
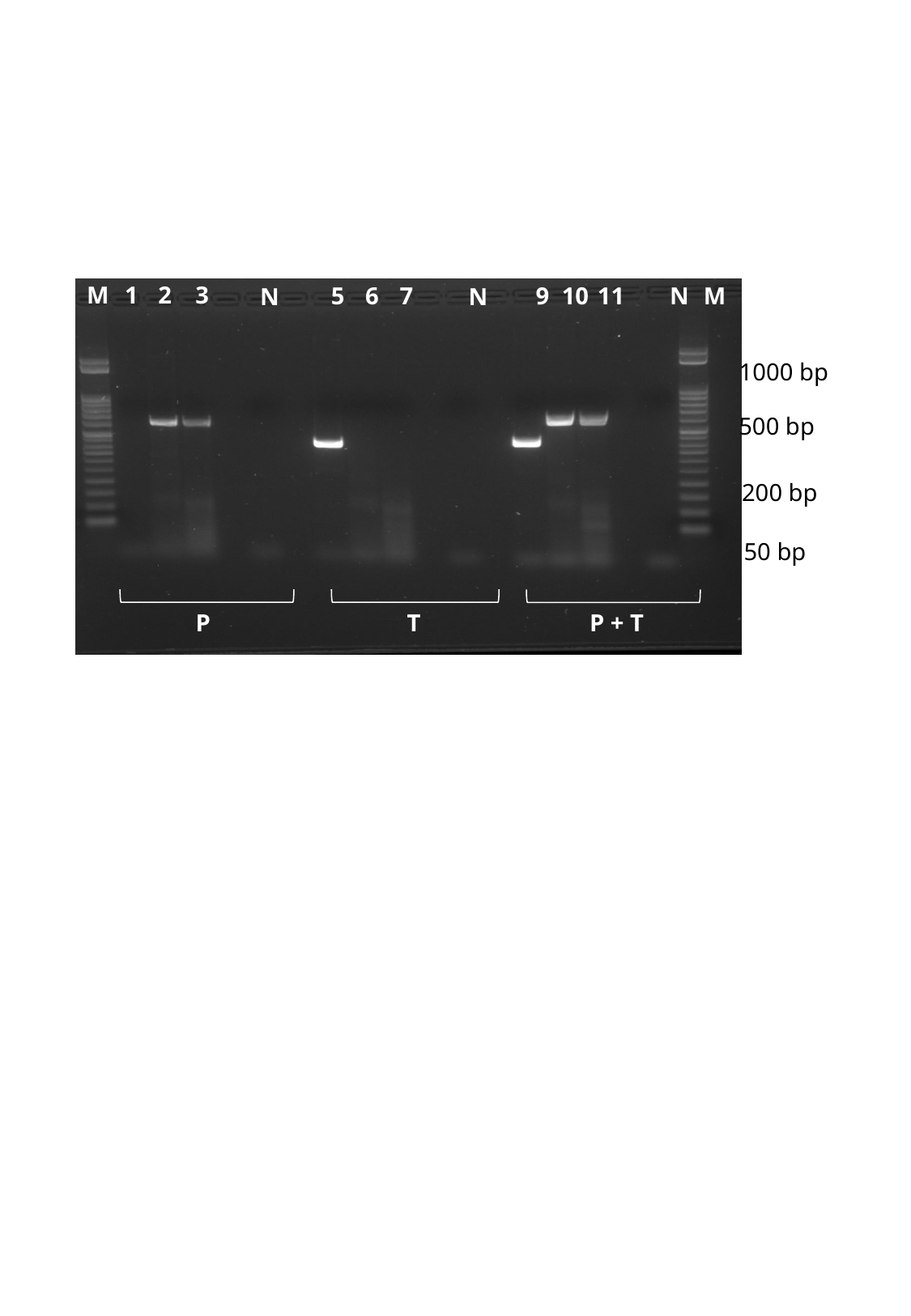

2
1
M
3
7
11
10
N
6
9
5
M
N
N
1000 bp
500 bp
200 bp
50 bp
P + T
T
P

Supplement: Supporting Information 2 — Figure S1: Agarose gel of amplified fragments using Cx. pipiens-specific primers (P), Cx. torrentium-specific primers (T), and a multiplex reaction combining all three primers (P+T). M = DNA stepladder, N = negative control, lane 1 = Cx. torrentium DNA not amplified with Cx. pipiens specific primers, lane 2 = Cx. pipiens DNA amplified with Cx. pipiens specific primers, lane 3 = WNV-positive mosquito pool amplified with Cx. pipiens specific primers, lane 5 = Cx. torrentium DNA amplified with Cx. torrentium-specific primers, lane 6 = Cx. pipiens DNA not amplified with Cx. torrentium-specific primers, lane 7 = WNV-positive mosquito pool not amplified with Cx. torrentium-specific primers, lane 9 = Cx. torrentium DNA amplified with multiplex reaction combining all three primers, lane 10 = Cx. pipiens DNA amplified with multiplex reaction combining all three primers, lane 11 = WNV-positive mosquito pool amplified with multiplex reaction combining all three primers. [file 3208806.f2.pptx]

## Slide 1
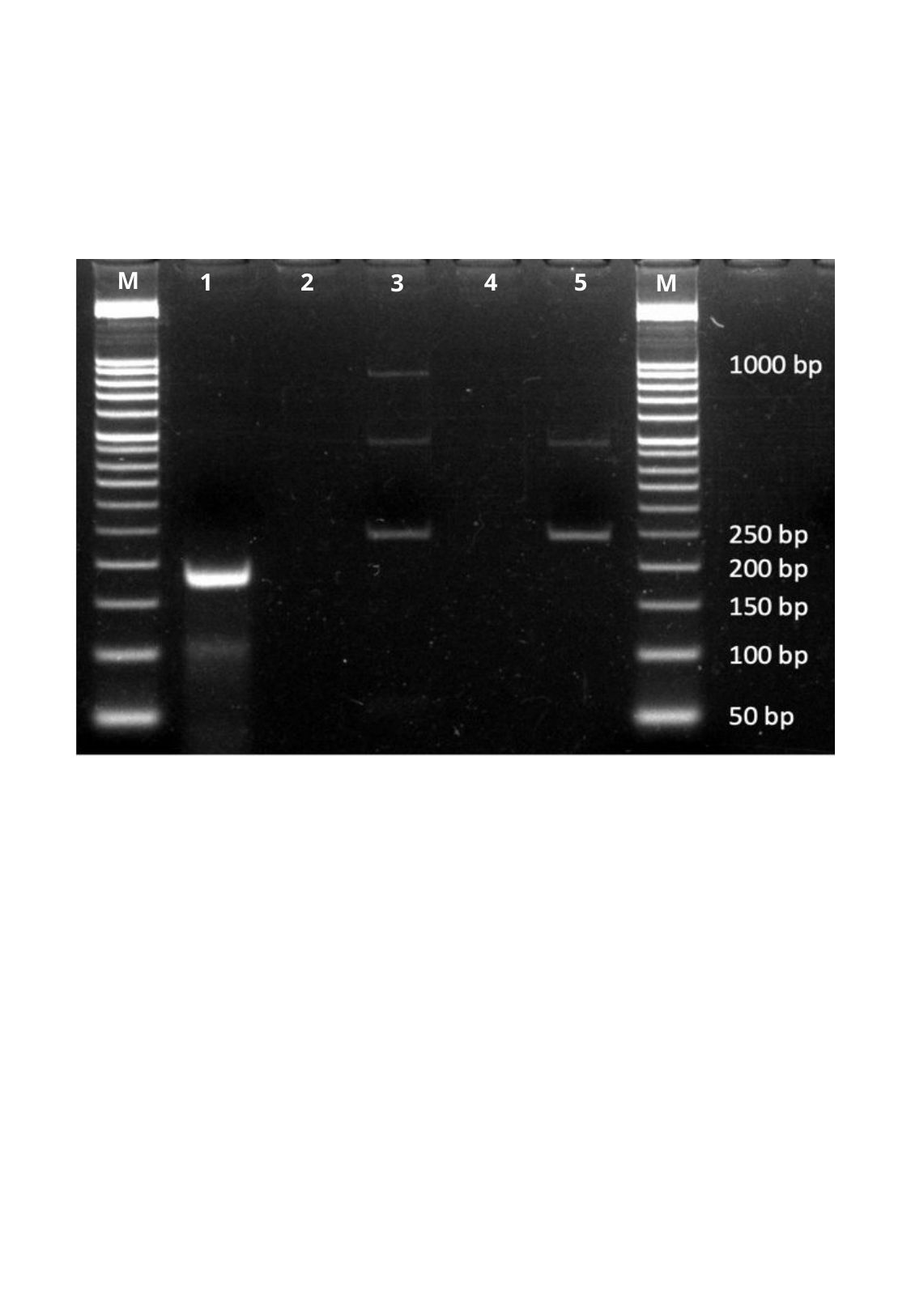

M
2
4
5
1
3
M

Supplement: Supporting Information 3 — Figure S2: Agarose gel of PCR discriminating between Cx. pipiens f. pipiens and Cx. pipiens f. molestus. M = DNA stepladder, lane 1 = WNV-positive mosquito pool showing a Cx. pipiens f. pipiens specific band (185 bp), lane 3 and 5 = Cx. pipiens f. molestus DNA showing a specific band (241 bp), lanes 2 and 4 = empty. [file 3208806.f3.pptx]
