# Supplementary material for: First Detection of West Nile Virus (WNV) Lineage 2 in Mosquitoes in the Republic of Kosovo
Source: Transbound Emerg Dis. 2025 Jun 24;2025:3208806. doi: 10.1155/tbed/3208806 (PMC12213049; doi:10.1155/tbed/3208806)
Supplement: Supporting Information 4 — Table S1: Sequence identity matrix comparing percent nucleotide (lower) and percent amino acid (upper) identity between the selected strains across the complete 10302 nt polyprotein open reading frame. [file 3208806.f4.docx]

**Supporting Information 4: Table S1.** Sequence identity matrix comparing percent nucleotide (lower) and percent amino acid (upper) identity between the selected strains across the complete 10302 nt polyprotein open reading frame.

| **NT\AA** | **DQ318019** | **EF429198** | **KC496015** | **KF179640** | **MZ190464** | **MZ190465** | **MZ190466** | **MZ190467** | **OP179287** | **PP212881** | **PQ053331** | **PQ435205** | **Kosovo_**  **2022** |
| --- | --- | --- | --- | --- | --- | --- | --- | --- | --- | --- | --- | --- | --- |
| **DQ318019** |  | 99.4 | 99.4 | 99.6 | 99.2 | 99.3 | 99.3 | 99.3 | 99.4 | 99.5 | 99.3 | 99.3 | 99.4 |
| **EF429198** | 97.9 |  | 99.2 | 99.4 | 99.0 | 99.1 | 99.0 | 99.1 | 99.1 | 99.2 | 99.0 | 99.0 | 99.2 |
| **KC496015** | 97.7 | 97.3 |  | 99.7 | 99.5 | 99.6 | 99.6 | 99.6 | 99.7 | 99.7 | 99.6 | 99.6 | 99.7 |
| **KF179640** | 97.8 | 97.5 | 99.6 |  | 99.5 | 99.6 | 99.6 | 99.6 | 99.7 | 99.8 | 99.6 | 99.6 | 99.7 |
| **MZ190464** | 97.4 | 97.0 | 99.3 | 99.3 |  | 99.4 | 99.9 | 99.9 | 99.5 | 99.6 | 99.4 | 99.4 | 99.5 |
| **MZ190465** | 97.5 | 97.2 | 99.5 | 99.5 | 99.2 |  | 99.4 | 99.5 | 99.5 | 99.6 | 99.4 | 99.4 | 99.6 |
| **MZ190466** | 97.4 | 97.0 | 99.3 | 99.3 | 99.8 | 99.3 |  | 100.0 | 99.6 | 99.7 | 99.5 | 99.5 | 99.6 |
| **MZ190467** | 97.5 | 97.1 | 99.5 | 99.4 | 99.9 | 99.4 | 99.9 |  | 99.6 | 99.7 | 99.5 | 99.5 | 99.7 |
| **OP179287** | 97.6 | 97.1 | 99.4 | 99.4 | 99.2 | 99.3 | 99.2 | 99.3 |  | 99.9 | 99.7 | 99.7 | 99.8 |
| **PP212881** | 97.5 | 97.1 | 99.4 | 99.3 | 99.2 | 99.2 | 99.2 | 99.3 | 99.7 |  | 99.8 | 99.8 | 99.9 |
| **PQ053331** | 97.5 | 97.0 | 99.3 | 99.3 | 99.1 | 99.2 | 99.1 | 99.3 | 99.6 | 99.7 |  | 99.6 | 99.7 |
| **PQ435205** | 97.4 | 96.9 | 99.2 | 99.2 | 99.0 | 99.1 | 99.1 | 99.2 | 99.5 | 99.7 | 99.5 |  | 99.7 |
| **Kosovo_2022** | 97.6 | 97.2 | 99.5 | 99.4 | 99.3 | 99.3 | 99.3 | 99.4 | 99.7 | 99.7 | 99.6 | 99.6 |  |
