# Supplementary material for: First Detection of West Nile Virus (WNV) Lineage 2 in Mosquitoes in the Republic of Kosovo
Source: Transbound Emerg Dis. 2025 Jun 24;2025:3208806. doi: 10.1155/tbed/3208806 (PMC12213049; doi:10.1155/tbed/3208806)
Supplement: Supporting Information 5 — Table S2: Percent nucleotide identity for each of the gene products made by posttranslational cleavage of the WNV lineage 2 polyprotein compared to a sequence identified in mosquitoes in Kosovo, 2022. [file 3208806.f5.docx]

**Supporting Information 5: Table S2.** Percent nucleotide identity for each of the gene products made by post-translational cleavage of the WNV lineage 2 polyprotein compared to a sequence identified in mosquitoes in Kosovo, 2022.

| **Accession** | **C** | **ancC** | **prM** | **pr** | **M** | **E** | **NS1** | **NS2a** | **NS2b** | **NS3** | **NS4a** | **2k** | **NS4b** | **NS5** |
| --- | --- | --- | --- | --- | --- | --- | --- | --- | --- | --- | --- | --- | --- | --- |
| **DQ318019** | 99.05% | 98.64% | 98.20% | 98.19% | 98.22% | 97.67% | 97.54% | 98.41% | 95.67% | 97.74% | 98.93% | 95.65% | 96.35% | 97.57% |
| **EF429198** | 98.73% | 98.10% | 98.40% | 98.19% | 98.67% | 97.21% | 97.44% | 97.26% | 96.95% | 97.15% | 98.13% | 94.20% | 96.22% | 97.02% |
| **KC496015** | 99.68% | 99.46% | 99.60% | 100% | 99.11% | 99.53% | 99.62% | 99.71% | 98.47% | 99.30% | 99.73% | 98.55% | 98.96% | 99.67% |
| **KF179640** | 99.68% | 99.46% | 99.60% | 99.64% | 99.56% | 99.53% | 99.53% | 99.57% | 98.47% | 99.25% | 100% | 98.55% | 99.09% | 99.52% |
| **MZ190464** | 99.05% | 98.64% | 99.00% | 99.28% | 98.67% | 99.33% | 99.72% | 99.57% | 98.47% | 99.03% | 99.47% | 100% | 99.35% | 99.30% |
| **MZ190465** | 99.68% | 99.46% | 99.40% | 99.64% | 99.11% | 99.27% | 99.24% | 99.42% | 98.22% | 99.19% | 100% | 98.55% | 99.48% | 99.56% |
| **MZ190466** | 99.05% | 98.64% | 99.00% | 99.64% | 98.22% | 99.33% | 99.91% | 99.57% | 98.47% | 99.08% | 99.73% | 100% | 99.22% | 99.26% |
| **MZ190467** | 99.37% | 98.92% | 99.20% | 99.64% | 98.67% | 99.47% | 99.91% | 99.71% | 98.47% | 99.14% | 99.73% | 100% | 99.35% | 99.41% |
| **PQ435205** | 100% | 99.73% | 100% | 100% | 100% | 99.73% | 99.72% | 98.99% | 98.98% | 99.35% | 99.20% | 100% | 99.87% | 99.63% |
| **PP212881** | 99.68% | 99.46% | 100% | 100% | 100% | 99.73% | 99.81% | 99.86% | 99.49% | 99.52% | 100% | 100% | 99.74% | 99.59% |
| **PQ053331** | 100% | 99.73% | 100% | 100% | 100% | 99.93% | 99.81% | 99.86% | 98.73% | 99.41% | 99.47% | 98.55% | 99.74% | 99.56% |
| **OP179287** | 100% | 99.73% | 100% | 100% | 100% | 99.80% | 99.81% | 99.86% | 99.24% | 99.52% | 99.47% | 100% | 99.87% | 99.85% |
