# Supplementary material for: First Detection of West Nile Virus (WNV) Lineage 2 in Mosquitoes in the Republic of Kosovo
Source: Transbound Emerg Dis. 2025 Jun 24;2025:3208806. doi: 10.1155/tbed/3208806 (PMC12213049; doi:10.1155/tbed/3208806)
Supplement: Supporting Information 6 — Table S3: Percent amino acid identity for each of the gene products made by post-translational cleavage of the WNV lineage 2 polyprotein compared to a sequence identified in mosquitoes in Kosovo, 2022. [file 3208806.f6.docx]

**Supporting Information 6: Table S3.** Percent amino acid identity for each of the gene products made by post-translational cleavage of the WNV lineage 2 polyprotein compared to a sequence identified in mosquitoes in Kosovo, 2022.

| **Accession** | **C** | **ancC** | **prM** | **pr** | **M** | **E** | **NS1** | **NS2a** | **NS2b** | **NS3** | **NS4a** | **2k** | **NS4b** | **NS5** |
| --- | --- | --- | --- | --- | --- | --- | --- | --- | --- | --- | --- | --- | --- | --- |
| **DQ318019** | 100% | 99.19% | 100% | 100% | 100% | 99% | 99.43% | 100% | 98.47% | 99.52% | 100% | 100% | 98.44% | 99.78% |
| **EF429198** | 100% | 99.19% | 100% | 100% | 100% | 99.6% | 99.15% | 99.57% | 98.47% | 99.52% | 100% | 100% | 98.44% | 98.67% |
| **KC496015** | 100% | 99.19% | 100% | 100% | 100% | 99.4% | 99.43% | 100% | 99.24% | 99.84% | 100% | 100% | 99.22% | 100% |
| **KF179640** | 100% | 99.19% | 100% | 100% | 100% | 99.6% | 99.72% | 100% | 99.24% | 99.68% | 100% | 100% | 99.22% | 100% |
| **MZ190464** | 100% | 98.37% | 99.4% | 100% | 98.67% | 99.4% | 99.72% | 100% | 100% | 99.35% | 99.2% | 100% | 100% | 99.56% |
| **MZ190465** | 100% | 99.19% | 99.4% | 98.91% | 100% | 99% | 99.43% | 100% | 98.47% | 99.84% | 100% | 100% | 99.61% | 99.89% |
| **MZ190466** | 100% | 98.37% | 99.4% | 100% | 98.67% | 99.6% | 99.72% | 100% | 100% | 99.35% | 100% | 100% | 100% | 99.67% |
| **MZ190467** | 100% | 98.37% | 99.4% | 100% | 98.67% | 99.8% | 99.72% | 100% | 100% | 99.35% | 100% | 100% | 100% | 99.67% |
| **PQ435205** | 100% | 99.19% | 100% | 100% | 100% | 100% | 99.72% | 98.7% | 99.24% | 99.68% | 100% | 100% | 100% | 100% |
| **PP212881** | 100% | 99.19% | 100% | 100% | 100% | 100% | 100% | 100% | 100% | 99.84% | 100% | 100% | 100% | 99.89% |
| **PQ053331** | 100% | 99.19% | 100% | 100% | 100% | 100% | 100% | 100% | 100% | 99.35% | 100% | 100% | 100% | 99.56% |
| **OP179287** | 100% | 99.19% | 100% | 100% | 100% | 100% | 99.43% | 100% | 100% | 99.84% | 99.2% | 100% | 99.61% | 100% |
