# Supplementary material for: First Detection of West Nile Virus (WNV) Lineage 2 in Mosquitoes in the Republic of Kosovo
Source: Transbound Emerg Dis. 2025 Jun 24;2025:3208806. doi: 10.1155/tbed/3208806 (PMC12213049; doi:10.1155/tbed/3208806)
Supplement: Supporting Information 7 — Table S4: Specific nonsynonymous mutations in the structural proteins (anchored capsid, “ancC”; membrane glycoprotein, “M”; envelope glycoprotein, “E”) of the selected West Nile virus lineage 2. [file 3208806.f7.docx]

**Supporting Information 7: Table S4.** Specific nonsynonymous mutations in the structural proteins (anchored capsid, “ancC”; membrane glycoprotein, “M”; envelope glycoprotein, “E”) of the selected West Nile virus lineage 2.

|  | **ancC** | | | **M** | **E** | | | | | | | | | | |
| --- | --- | --- | --- | --- | --- | --- | --- | --- | --- | --- | --- | --- | --- | --- | --- |
| **Accession** | **114** | **121** | **124** | **274** | **381** | **444** | **449** | **530** | **603** | **629** | **668** | **689** | **726** | **729** | **777** |
| **DQ318019** | L | A | V | Q | V | - | I | V | R | P | P | K | T | G | V |
| **EF429198** | L | A | V | Q | V | NYST | I | M | G | P | P | K | T | G | V |
| **KC496015** | L | A | V | Q | V | NYST | I | M | G | P | P | K | T | G | V |
| **KF179640** | L | A | V | Q | V | NYST | I | M | G | P | P | K | T | G | V |
| **MZ190464** | M | A | V | P | V | NYST | K | M | G | H | P | K | T | G | V |
| **MZ190465** | L | A | L | Q | L | NYST | I | M | G | P | S | K | A | G | V |
| **MZ190466** | M | A | V | P | V | NYST | T | M | G | P | P | K | T | A | V |
| **MZ190467** | M | A | V | P | V | NYST | T | M | G | P | P | K | T | G | V |
| **OP179287** | L | A | V | Q | V | NYST | T | M | G | P | P | R | T | G | V |
| **PP212881** | L | A | V | Q | V | NYST | T | M | G | P | P | R | T | G | V |
| **PQ053331** | L | A | V | Q | V | NYST | T | M | G | P | P | R | T | G | V |
| **PQ435205** | L | A | V | Q | V | NYST | T | M | G | P | P | R | T | G | A |
| **This study** | L | V | V | Q | V | NYST | T | M | G | P | P | R | T | G | V |
