# Supplementary material for: First Detection of West Nile Virus (WNV) Lineage 2 in Mosquitoes in the Republic of Kosovo
Source: Transbound Emerg Dis. 2025 Jun 24;2025:3208806. doi: 10.1155/tbed/3208806 (PMC12213049; doi:10.1155/tbed/3208806)
Supplement: Supporting Information 8 — Table S5: Specific nonsynonymous mutations in the nonstructural proteins (NS1, NS2a, NS2b) of the selected West Nile virus lineage 2. [file 3208806.f8.docx]

**Supporting Information 8: Table S5.** Specific nonsynonymous mutations in the non-structural proteins (NS1, NS2a, NS2b) of the selected West Nile virus lineage 2.

|  | **NS1** | | | | | | | | **NS2a** | | | **NS2b** | | | | | |
| --- | --- | --- | --- | --- | --- | --- | --- | --- | --- | --- | --- | --- | --- | --- | --- | --- | --- |
| **Accession** | **820** | **835** | **837** | **882** | **900** | **990** | **1035** | **1066** | **1196** | **1241** | **1255** | **1405** | **1416** | **1462** | **1490** | **1493** | **1502** |
| **DQ318019** | M | K | I | K | K | S | I | I | V | R | A | I | A | M | L | V | Y |
| **EF429198** | I | K | I | K | K | S | I | I | V | K | A | I | A | M | L | V | Y |
| **KC496015** | M | R | I | K | K | A | I | I | V | R | A | V | A | I | L | I | Y |
| **KF179640** | M | R | I | K | K | S | I | I | V | R | A | I | A | I | L | V | Y |
| **MZ190464** | M | R | I | K | K | S | I | I | V | R | A | I | A | I | L | I | Y |
| **MZ190465** | M | R | I | N | K | S | I | I | V | R | A | I | S | I | P | I | Y |
| **MZ190466** | M | R | I | K | K | S | I | I | V | R | A | I | A | I | L | I | Y |
| **MZ190467** | M | R | I | K | K | S | I | I | V | R | A | I | A | I | L | I | Y |
| **OP179287** | M | R | I | K | R | S | V | L | V | R | A | I | A | I | L | I | Y |
| **PP212881** | M | R | I | K | K | S | V | I | V | R | A | I | A | I | L | I | Y |
| **PQ053331** | M | R | I | K | K | S | V | I | V | R | A | I | A | I | L | I | Y |
| **PQ435205** | M | R | S | K | K | S | V | I | D | R | V | I | A | I | L | I | C |
| **This study** | M | R | I | K | K | S | V | I | V | R | A | I | A | I | L | I | Y |
