# Supplementary material for: First Detection of West Nile Virus (WNV) Lineage 2 in Mosquitoes in the Republic of Kosovo
Source: Transbound Emerg Dis. 2025 Jun 24;2025:3208806. doi: 10.1155/tbed/3208806 (PMC12213049; doi:10.1155/tbed/3208806)
Supplement: Supporting Information 9 — Table S6: Specific nonsynonymous mutations in the non-structural proteins (NS3, NS4a, NS4b) of the selected West Nile virus lineage 2. [file 3208806.f9.docx]

**Supporting Information 9: Table S6.** Specific nonsynonymous mutations in the non-structural proteins (NS3, NS4a, NS4b) of the selected West Nile virus lineage 2.

|  | **NS3** | | | | | | | | | | **NS4a** | | **NS4b** | | | | | | | |
| --- | --- | --- | --- | --- | --- | --- | --- | --- | --- | --- | --- | --- | --- | --- | --- | --- | --- | --- | --- | --- |
| **Accession** | **1516** | **1519** | **1547** | **1574** | **1720** | **1754** | **1787** | **1906** | **2026** | **2087** | **2149** | **2198** | **2284** | **2287** | **2293** | **2296** | **2305** | **2322** | **2386** | **2515** |
| **DQ318019** | K | K | V | W | K | H | I | D | D | V | D | R | N | G | H | A | S | T | V | M |
| **EF429198** | K | K | V | W | K | H | I | D | D | V | D | R | N | S | K | A | S | T | V | T |
| **KC496015** | R | K | V | W | K | P | I | D | D | V | D | R | S | G | R | T | N | A | V | T |
| **KF179640** | R | K | V | W | K | H | I | D | D | V | D | R | N | S | K | T | N | T | V | T |
| **MZ190464** | R | K | V | W | R | P | I | E | D | I | E | R | N | G | K | T | N | A | V | T |
| **MZ190465** | R | K | V | W | K | P | I | D | D | V | D | R | N | G | K | T | N | A | M | T |
| **MZ190466** | R | K | V | W | R | P | I | E | D | I | D | R | N | G | K | T | N | A | V | T |
| **MZ190467** | R | K | V | W | R | P | I | E | D | I | D | R | N | G | K | T | N | A | V | T |
| **OP179287** | R | K | V | W | K | P | I | D | D | V | D | S | D | G | K | T | N | A | V | T |
| **PP212881** | R | K | V | W | K | P | I | D | D | V | D | R | N | G | K | T | N | A | V | T |
| **PQ053331** | R | Q | G | L | K | P | I | D | D | V | D | R | N | G | K | T | N | A | V | T |
| **PQ435205** | R | K | V | W | K | P | I | D | A | V | D | R | N | G | K | T | N | A | V | T |
| **This study** | R | K | V | W | K | P | V | D | D | V | D | R | N | G | K | T | N | A | V | T |
