# Supplementary material for: First Detection of West Nile Virus (WNV) Lineage 2 in Mosquitoes in the Republic of Kosovo
Source: Transbound Emerg Dis. 2025 Jun 24;2025:3208806. doi: 10.1155/tbed/3208806 (PMC12213049; doi:10.1155/tbed/3208806)
Supplement: Supporting Information 10 — Table S7: Specific nonsynonymous mutations in the RNA-dependent RNA polymerase (NS5) of selected West Nile virus lineage 2. [file 3208806.f10.docx]

**Supporting Information 10: Table S7.** Specific nonsynonymous mutations in the RNA-dependent RNA polymerase (NS5) of selected West Nile virus lineage 2.

|  | **NS5** | | | | | | | | | | | | | | | | | |
| --- | --- | --- | --- | --- | --- | --- | --- | --- | --- | --- | --- | --- | --- | --- | --- | --- | --- | --- |
| **Accession** | **2544** | **2690** | **2719** | **2747** | **2988** | **3055** | **3061** | **3072** | **3096** | **3111** | **3118** | **3143** | **3145** | **3154** | **3173** | **3192** | **3257** | **3425** |
| **DQ318019** | R | T | K | EMY | K | T | VY | R | A | V | G | T | T | MM | K | M | T | DT |
| **EF429198** | R | T | K | EMY | K | I | IF | P | A | G | R | P | S | RR | N | M | T | DA |
| **KC496015** | R | T | R | EMY | K | T | IY | R | A | V | G | T | T | MM | K | M | T | DT |
| **KF179640** | R | T | R | EMY | K | T | IY | R | A | V | G | T | T | MM | K | M | T | DT |
| **MZ190464** | K | T | R | EMY | N | T | IY | R | A | V | G | T | T | MM | K | L | T | ET |
| **MZ190465** | R | T | R | EMY | K | T | IY | R | P | V | G | T | T | MM | K | M | T | DT |
| **MZ190466** | K | T | R | EMY | N | T | IY | R | A | V | G | T | T | MM | K | L | T | DT |
| **MZ190467** | K | T | R | EMY | N | T | IY | R | A | V | G | T | T | MM | K | L | T | DT |
| **OP179287** | R | T | R | EMY | K | T | IY | R | A | V | G | T | T | MM | K | M | T | DT |
| **PP212881** | R | S | R | EMY | K | T | IY | R | A | V | G | T | T | MM | K | M | T | DT |
| **PQ053331** | R | T | R | GIC | K | T | IY | R | A | V | G | T | T | MM | K | M | M | DT |
| **PQ435205** | R | T | R | EMY | K | T | IY | R | A | V | G | T | T | MM | K | M | T | DT |
| **This study** | R | T | R | EMY | K | T | IY | R | A | V | G | T | T | MM | K | M | T | DT |
